# Prediction of Conversion from Mild Cognitive Impairment to Alzheimer's Disease Using MRI and Structural Network Features

**DOI:** 10.3389/fnagi.2016.00076

**Published:** 2016-04-19

**Authors:** Rizhen Wei, Chuhan Li, Noa Fogelson, Ling Li

**Affiliations:** ^1^Key Laboratory for NeuroInformation of Ministry of Education, High-Field Magnetic Resonance Brain Imaging Key Laboratory of Sichuan Province, Center for Information in Medicine, School of Life Science and Technology, University of Electronic Science and Technology of ChinaChengdu, China; ^2^School of Computer Science and Engineering, University of Electronic Science and Technology of ChinaChengdu, China; ^3^EEG and Cognition Laboratory, University of A CoruñaA Coruña, Spain

**Keywords:** mild cognitive impairment, MRI, structural network, prediction, early detection

## Abstract

Optimized magnetic resonance imaging (MRI) features and abnormalities of brain network architectures may allow earlier detection and accurate prediction of the progression from mild cognitive impairment (MCI) to Alzheimer's disease (AD). In this study, we proposed a classification framework to distinguish MCI converters (MCIc) from MCI non-converters (MCInc) by using a combination of FreeSurfer-derived MRI features and nodal features derived from the thickness network. At the feature selection step, we first employed sparse linear regression with stability selection, for the selection of discriminative features in the iterative combinations of MRI and network measures. Subsequently the top *K* features of available combinations were selected as optimal features for classification. To obtain unbiased results, support vector machine (SVM) classifiers with nested cross validation were used for classification. The combination of 10 features including those from MRI and network measures attained accuracies of 66.04, 76.39, 74.66, and 73.91% for mixed conversion time, 6, 12, and 18 months before diagnosis of probable AD, respectively. Analysis of the diagnostic power of different time periods before diagnosis of probable AD showed that short-term prediction (6 and 12 months) achieved more stable and higher AUC scores compared with long-term prediction (18 months), with *K*-values from 1 to 30. The present results suggest that meaningful predictors composed of MRI and network measures may offer the possibility for early detection of progression from MCI to AD.

## Introduction

Mild cognitive impairment (MCI), commonly characterized by slight cognitive deficits but largely intact activities of daily living (Petersen, 2004), is a transitional stage between the healthy aging and dementia. Several studies have suggested that individuals with MCI tend to progress to Alzheimer's disease (AD) at a rate of approximately 10–15% per year (Hänninen et al., 2002; Grundman et al., 2004), while normal controls (NC) develop dementia at a lower rate of 1–2% per year (Bischkopf et al., 2002). In these studies, conversion was considered over the course of 6 months up to a 4-year follow-up period. MCI remains challenging for diagnosis due to the mild symptoms of cognitive impairment, various etiologies and pathologies, and high rates of reversion back to normal. Thus, early detection of MCI individuals who are suffering from a high risk of conversion from MCI to AD is of increasing clinical importance in potentially delaying or preventing the transition from MCI to AD.

Magnetic resonance imaging (MRI) techniques have provided an efficient and non-invasive way to delineate brain atrophy. Recently, several studies have demonstrated that cortical thickness and subcortical volumetry/shape derived from baselines MRI scans can detect patterns of cerebral atrophy in AD (Fan et al., 2008; Lerch et al., 2008; Vemuri et al., 2008; Frisoni et al., 2010; Julkunen et al., 2010), but with that these have limited prediction accuracy of the conversion to AD in MCI patients (Risacher et al., 2009; Cuingnet et al., 2011). The limited sensitivity of MRI biomarkers in predicting the conversion of MCI subjects has prompted researchers to evaluate the combined prognostic value of different biomarkers. Recent findings (Cui et al., 2011; Gomar et al., 2011; Ewers et al., 2012; Westman et al., 2012; Liu et al., 2014) show that the combination of a range of different biomarkers have better predictive power compared with a single biomarker. However, collecting multi-modality data at the same time may not be applicable in practice.

In addition to the raw features obtained from MRI, structural brain network measures, referred to as the anatomical connection pattern between different neuronal elements (He et al., 2009; Jie et al., 2014; Li and Zhao, 2015), provide new insights into the network organization, topology, and complex dynamics of the brain, as well as further understanding of the pathogenesis of neurological disorders (Bullmore and Sporns, 2009; Zalesky et al., 2010). Abnormalities of structural networks have been observed in AD and MCI patients (Stam et al., 2007; He et al., 2008; Yao et al., 2010; Tijms et al., 2013; Zhou and Lui, 2013). Yao and colleagues used thickness cortical networks to study the aberrant brain structures in MCI and report that the nodal centrality in MCI, compared with a NC group, showed decreases in the left lingual gyrus, middle temporal gyrus (MTG), and increases in the precuneus cortex (Yao et al., 2010). Zhou and Lui (2013) also used cortical thickness to detect small-world properties alteration in MCI and reported that MCI converters (MCIc) showed the lowest local efficiency during the conversion period to AD; while the MCI non-converters (MCInc) showed the highest local and global efficiency.

These approaches which used optimized MRI features achieve encouraging accuracies (over 60%). However, few studies analyzed the co-variation of abnormalities in different regions of interest (ROIs), which can be characterized by network patterns and could contribute to reliable and sensitive classification (Dai et al., 2013). Indeed, the pattern of AD pathology is complex and evolves as disease progresses (Fan et al., 2008) and many regions share similar patterns of abnormal brain morphometric. Thus, informative network topology may be potentially useful for classification. In addition, many factors such as the heterogeneity of the MRI images (Eskildsen et al., 2013) and the imbalanced data between groups (Johnstone et al., 2012; Dubey et al., 2014) can also lead to overestimations.

The main objective of the current study was to determine whether the combined use of structural brain measures and thickness network alterations, may improve the accuracy and the sensitivity in identifying prodromal AD. To this end, we proposed a classification framework to distinguish MCIc from MCInc by using a combination of features from FreeSurfer-derived MRI features and nodal parameters derived from thickness network. To obtain predictive nodal information for each individual, we first established a weight network by using a kernel function and then thresholded it to a binary network. Finally, nodal properties were measured at a high discriminative connection cost. At the feature selection step, we first employed sparse linear regression with stability selection for robust feature selection in the iterative combination of MRI and network measures, and then top *K* features of available combinations were selected as optimal features for classification. To obtain unbiased results, support vector machine (SVM) classifiers with nested cross validation were used for classification. The secondary goal of this study was to measure the impact of different conversion time periods before diagnosis of probable AD, and to evaluate different predictive values between two groups. To that purpose, we homogenized the MCIc images with respect to “time to conversion.” Thus, MCIc patients were subdivided into four groups: mixed for baseline, 6, 12, and 18 months before diagnosis of probable AD. Our hypothesis was that network topological measures might be potentially useful for classification of imminent conversion, and the effective combination of brain morphometric and thickness network measures may improve the prediction of conversion from MCI to AD. Besides, more stable and higher classification accuracy could be obtained for the short-term prediction (6 and 12 months) compared with the long-term prediction (18 months).

## Materials and methods

### Participants

Data used in this article were obtained from the Alzheimer's Disease Neuroimaging Initiative (ADNI) database (adni.loni.usc.edu). The ADNI was launched in 2003 as a public-private partnership, led by Principal Investigator Michael W. Weiner, MD. The primary goal of ADNI has been to test whether serial MRI, positron emission tomography (PET), other biological markers, and clinical and neuropsychological assessment can be combined to measure the progression of MCI and early Alzheimer's disease (AD).

The eligibility criteria for inclusion of subjects are described at: http://adni.loni.usc.edu/wp-content/uploads/2010/09/ADNI_GeneralProceduresManual.pdf. General criteria for MCI were as follows: (1) Mini-Mental-State-Examination (MMSE) scores between 24 and 30 (inclusive), (2) a memory complaint, objective memory loss measured by education adjusted scores on the Wechsler Memory Scale Logical Memory II, (3) a Clinical Dementia Rating (CDR) of 0.5, and (4) absence of significant levels of impairment in other cognitive domains, essentially preserved activities of daily living, and an absence of dementia.

Several studies, which rendered the MCI converters with respect to “time to conversion,” have used baseline MRI scans to predict the conversion, since the MCI patients could convert anytime over the course of 6 months to 4 years. We categorized the MCI patients into converters and non-converters as in Wolz et al. (2011), where non-converters were defined as those that did not have a change of diagnosis within 36 months and the complementary MCI patients constituted the MCIc group. To assess the diagnostic power of different time periods before diagnosis of probable AD, we selected scans at various intervals prior to diagnosis. We selected MCIc scans at 6 (MCIc_m6), 12 (MCIc_m12), and 18 months (MCIc_m18) prior to AD diagnosis. MCIc scans at 24 and 36 months prior to AD diagnosis were excluded from the analysis due to the small samples and large imbalances between the two groups. To evaluate our method in comparison with the method using baseline scans for prediction, we also selected MCIc baseline data (MCIc_mixed) for prediction. Table 1 summarizes the selected MCI patients in our study.

**Table 1:** **Subject characteristics**.

	**MCIc_mixed**	**MCIc_m6**	**MCIc_m12**	**MCIc_m18**	**MCInc**	***P*-value**
Gender(F/M)	30/46	25/36	26/37	16/26	29/54	NS
Age	73.6 ± 7.8	74.5 ± 7.5	74.0 ± 7.8	74.3 ± 7.6	74.1 ± 7.3	NS
Education	15.8 ± 3.1	15.6 ± 3.1	15.9 ± 2.8	15.8 ± 2.9	15.8 ± 3.0	NS
CDR-SB	1.7 ± 1.1^a^	2.5 ± 1.2^a^	2.1 ± 1.1^a^	1.8 ± 1.0^a^	1.3 ± 0.6	*p* < 0.001
MMSE	26.5 ± 1.6	25.2 ± 2.5^a^	26.1 ± 2.1	25.9 ± 2.2	27.5 ± 1.7	*p* = 0.015

*Values represent mean ± SD. CDR-SB, Clinical Dementia Rating Sum of Boxes; MMSE, Mini Mental State Examination. Chi-square was used for gender comparison. A two-way student t-test was used for age, education, and neuropsychological test comparisons. NS, not significant*.

a *Indicates significance compared to the MCInc group*.

### MRI imaging acquisition

All scans used in the study were T1-weighted MPRAGE images acquired in 1.5-Tesla MR imaging instruments using a standardized protocol (Jack et al., 2008). Pre-processing images were downloaded from the public ADNI site (adni.loni.usc.edu). The images were preprocessed according to a number of steps detailed in the ADNI website, which contained (1) grad warp correction of image geometry distortion due to gradient non-linearity, (2) B1 non-uniformity processing to correct the image intensity non-uniformity, and (3) N3 processing to reduce residual intensity non-uniformity.

### Feature extraction

#### MRI features

**Figure 1:**
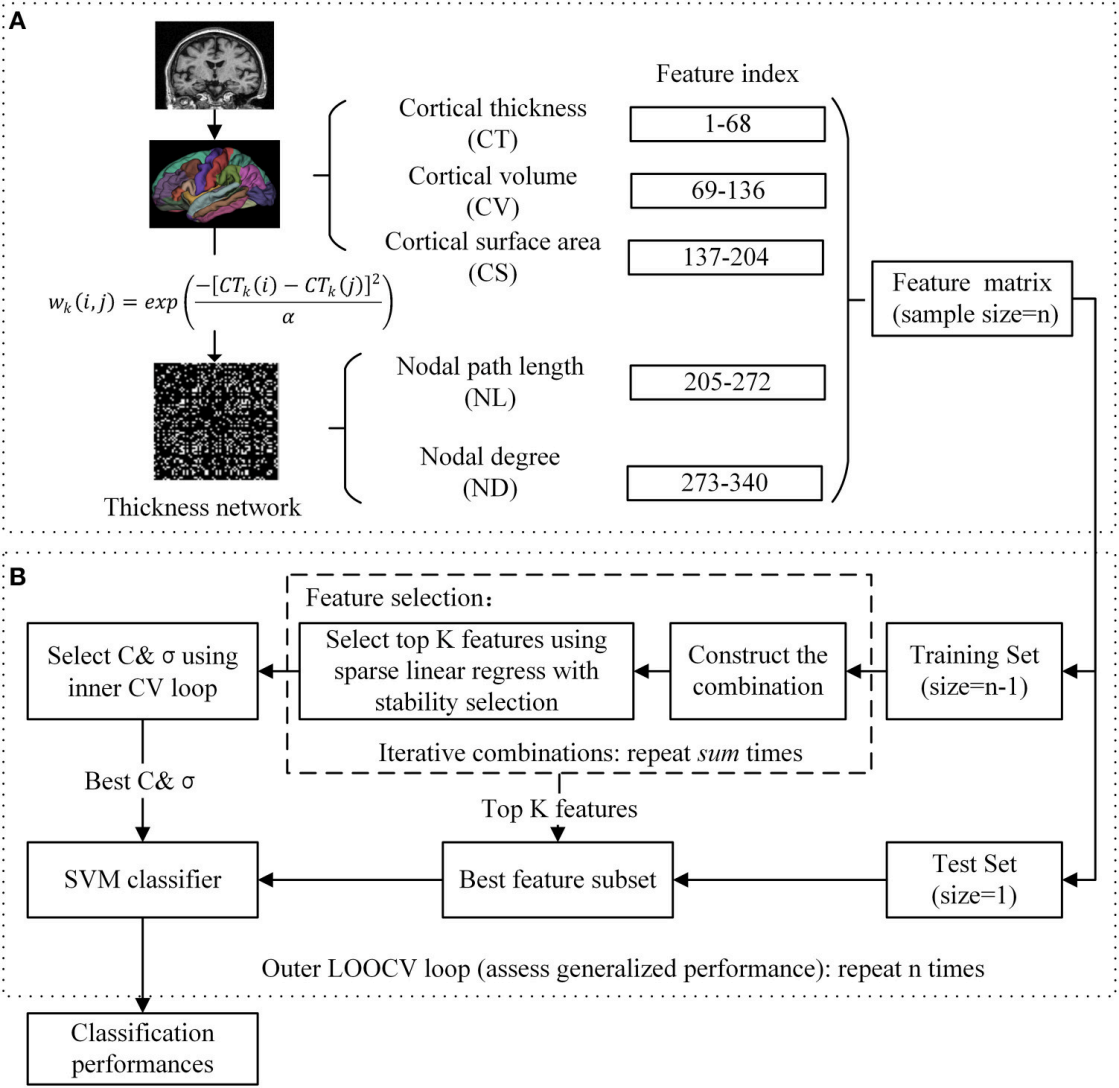
**Proposed prediction framework. (A)** Feature extraction: T1-weigthed images are processed and individual thickness network is constructed based on the difference in cortical thickness of a pair of ROIs. **(B)** Classification: SVM classifier with nested cross validation is implemented for classification.

The FreeSurfer 5.30 software package was utilized for cortical reconstruction and volumetric segmentation (FreeSurfer v5.30, http://surfer.nmr.mgh.harvard.edu/fswiki). In brief, the processing contains automated Talairach spaces transformation, intensity inhomogeneity correction, removal of non-brain tissue, intensity normalization, tissue segmentation (Fischl et al., 2002), automated topology correction, surface deformation to generate the gray/white matter boundary and gray matter/ Cerebrospinal Fluid (CSF) boundary, and parcellation of the cerebral cortex (Desikan et al., 2006). The quality of the raw MRI images, Talairach registration, intensity normalization, brain segmentation, and surface demarcation were assessed using a manual inspection protocol. The images that failed the stages of quality assurance were removed from subsequent analysis. The atlas used in FreeSurfer included 34 cortical ROIs per hemisphere (Table 2). For each cortical ROI, cortical thickness (CT), cortical volume (CV), and cortical surface area (CS) were calculated as three subtypes of MRI features. CT at each vertex of the cortex was calculated as the average shortest distance between white and pail surfaces. CS was calculated by computing the area of every triangle in a standardized spherical surface tessellation. CV at each vertex was computed by the product of the CS and CT at each surface vertex. This yielded a total of 204 cortical features for each subject (Figure 1A).

**Table 2:** **Anatomical regions**.

**Anatomical region**	**Abbreviation**	**Anatomical region**	**Abbreviation**
Banks superior temporal sulcus	BSTS	Pars Orbitalis	PORB
Caudal anterior cingulate cortex	cACC	Pars Triangularis	PTri
Caudal middle frontal gyrus	cMFG	Pericalcarine cortex	PCAL
Cuneus cortex	CUN	Postcentral gyrus	PoCG
Entorhinal cortex	ENT	Posterior cingulate cortex	PCC
Fusiform gyrus	FG	Precentral gyrus	PreCG
Inferior parietal cortex	IPC	Precuneus cortex	PCUN
Inferior temporal gyrus	ITG	Rostral anterior cingulate cortex	rACC
Isthmus of cingulate cortex	IstCC	Rostral middle frontal gyrus	rMFG
Lateral occipital cortex	LOC	Superior frontal gyrus	SFG
Lateral orbital frontal cortex	ORBlat	Superior parietal cortex	SPC
Lingual gyrus	LING	Superior temporal gyrus	STG
Medial orbital frontal cortex	ORBmid	Supramarginal gyrus	SMG
Middle temporal gyrus	MTG	Frontal pole	FP
Parahippocampal gyrus	PHG	Temporal pole	TP
Paracentral lobule	PCL	Transverse temporal cortex	TTC
Pars Opercularis	POperc	Insula	INS

#### Thickness network features

Similar to a prior study (Dai et al., 2013), the thickness network matrix *W*_*ij*_(*i, j* = 1, 2, …, *N, here N*= 68) for each individual was obtained by calculating the difference in cortical thickness between each pair of regions, and measured using the following kernel, with the weight defined as:

(1) $$\begin{array}{l} w_{k}\left(i,j\right)=exp\left(\frac{-{\left[{CT}_{k}\left(i\right)-{CT}_{k}\left(j\right)\right]}^{2}}{\alpha }\right) \end{array}$$

where *CT*_*k*_(*i*) represents the cortical thickness of *i* ROI of *k* subjects, and the kernel width α is 0.01. To simplify the statistical calculation, the thickness network matrix of each individual was thresholded into a binary matrix *B*_*ij*_ = [*b*_*ij*_], where the *b*_*ij*_ was 1 if the weight of the two ROIs was larger than the given threshold, and 0 otherwise. The threshold represents the network connection cost, defined as the ratio of the supra-threshold connections relative to the total possible number of connections in the network (Fornito et al., 2010). After applying each threshold, these binary matrices were then used as a basis for the network construction and graph analysis. We analyzed the full range of costs from 8 to 40%, at 1% intervals. The nodal properties were then extracted at a connection cost of 18%, at which the clustering coefficient showed the largest difference between the MCIc_mixed and MCInc groups. Finally, 136 nodal features including nodal path length (NL) and nodal degree (ND) were employed for subsequent analysis (Figure 1A). In brief, for a given node *i*, nodal path length and nodal degree were defined as follows:

(2) $$\begin{array}{c} L_{i}=\frac{\sum_{j\neq i\in V}L_{ij}}{\left(V-1\right)} \end{array}$$

(3) $$\begin{array}{r} k_{i}=\sum_{j\in V}b_{ij} \end{array}$$

where *L*_*ij*_ refers to the minimum number of edges between node pairs *i* and *j*, *V* is the size of a graph, and *b*_*ij*_ is the connection status between the node pairs *i* and *j*. Intuitively, path length *L*_*i*_ measures the speed of the message that passes through a given node, and the degree of an individual node *k*_*i*_ is equal to the number of links connected to that node, thus reflecting the level of interaction in the network.

### Feature selection

In the current study, as shown in Figure 1, we evaluated 340 features from five different categories (three types of MRI features and two types of network features) for each subject. We implemented the combination in an iterative manner to avoid making an arbitrary choice of the combination. Features were combined in every possible combination. The iteration pattern was described as follows:

(4) $$\begin{array}{l} sum=\sum_{i\;=\;1}^{i\;=\;5}C_{5}^{i} \end{array}$$

where *i* refers to the type of features, *sum* refers to the number of total iterative models. A total of 31 combinations were obtained for each diagnostic pair.

In each combination, we applied sparse linear regression for features selection using the L_1_-norm regularization (Tibshirani, 1996). Let $X={\left[x_{1},x_{2},\ldots ,x_{n}\right]}^{T}\in {\mathbb{R}}^{n\times m}$be a *n*×*m* matrix that represents *m* features of *n* samples, $y={\left[y_{1},y_{2},\ldots ,y_{n}\right]}^{T}\in {\mathbb{R}}^{n\times 1}$ be a *n* dimensional corresponding classification labels (*y*_*i*_ = 1 for MCIc and *y*_*i*_ = −1 for MCInc). The linear regression model was defined as follows:

(5) $$\hat{y}=Xw$$

where $w\;=\;{\left[w_{1},w_{2},\ldots ,w_{m}\right]}^{T}\in {\mathbb{R}}^{m\times 1}$ and $\hat{y}$ denotes the regression coefficient vector and the predicted label vector. One approach is to estimate the *w* by minimizing the following objective function:

(6) $$\underset{w}{\min}\frac{1}{2}\|Xw-y{\|}_{2}^{2}+\lambda \|w{\|}_{1}$$

where λ > 0 is a regularization parameter which controls the sparsity of the model, i.e., many of the entries of *w* are zero, and ||*w*||_1_ is the L_1_-norm of *w*, which is defined as $\overset{m}{\underset{i=1}{\sum }}|w_{i}|$. In this study, the SLEP package (Liu et al., 2009) was used for solving sparse linear regression. To address the problem of proper regularization we applied the stability selection using subsampling or bootstrapping (Meinshausen and Bühlmann, 2010) for robust feature selection. For each combination, we selected the top *K* (*K* = 10) features for subsequent analysis. After feature selection of each combination, the likelihood L for a feature index being selected in the combinations was calculated as follows:

(7) $$\begin{array}{l} L\left(l\right)=\frac{1}{sum}\sum_{i=1}^{sum}{sf}_{i}\left(l\right),where\;sf\left(l\right)=\{\begin{array}{c} 1,if\;selected\\ 0,otherwise \end{array} \end{array}$$

where *sum* is the number of combinations, *l* is the features index and *sf* is a binary function determining if *l* is selected in a combination. L is an expression of how often a feature is included among all combinations. Finally, the top K features were selected for classification.

### Classification

For the selected features, the SVM classifier was implemented using the LIBSVM toolbox (Chang and Lin, 2011), with radial basis function (RBF) and an optimal value for the penalized coefficient *C* (a constant determining the tradeoff between training error and model flatness). The RBF kernel was defined as follows:

(8) $$\begin{array}{l} K\left(x_{1},x_{2}\right)=exp\left(-\frac{\left||x_{1}-x_{2}|\right|}{2\sigma^{2}}\right) \end{array}$$

where *x*_1_, *x*_2_ are the two feature vectors and σ controls the width of the RBF kernel. In order to obtain an unbiased estimation and select the optimal SVM model, a nested cross validation (CV) was employed. For a training set, we selected the optimal hyperparameters (*C* and σ) through a grid-search and a 10-fold CV (inner CV). The outer CV that we used was the leave-one-out cross validation (LOOCV). In each fold of the outer CV, one sample was kept out for validation and the remaining were used for feature selection and training the classifier; then the performance of the training classifier was evaluated using the held-out sample. This run was repeated until all the subjects were excluded. The pipeline of our classification framework is presented in Figure 1. To evaluate the quality of the classification, we report four established measures: accuracy, sensitivity, specificity, and area under the curve (AUC). These measures were defined as follows:

(9) $$\begin{array}{r} Accuracy=\frac{TP+TN}{TP+TN+FP+FN},Sensitivity=\frac{TP}{TP+FN},\\ Specificity=\frac{TN}{TN+FP} \end{array}$$

where TP, TN, FP, FN denote true positive, true negative, false positive, and false negative, respectively. Following a common convention, we considered a correctly predicted MCIc as a true positive.

## Results

**Figure 2:**
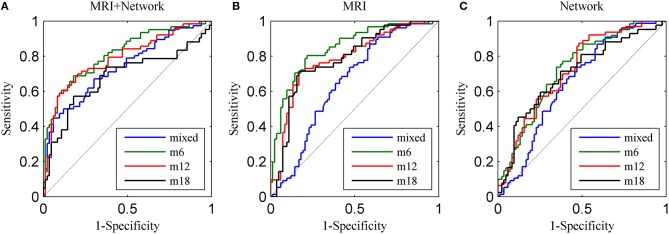
**ROC curves for the four diagnostic pairs using (A) top 10 combined features, (B) top 10 MRI features, and (C) top 10 network features**.

The LOOCV results of classification and receiver operating characteristic curves (ROCs) are depicted in Table 3 and Figure 2A. For the MCInc vs. MCIc_mixed model, the proposed method achieved a classification accuracy of 66.04% (sensitivity = 55.26%, specificity = 75.90%, AUC = 0.7346). For classifying MCIc_m6 from MCInc, combining the MRI with network measures, resulted in a higher accuracy of 76.39% (sensitivity = 65.57%, specificity = 84.34%, AUC = 0.8130). Specifically, we obtained slightly lower levels of accuracies for 12 and 18 months (74.66 and 73.91%, respectively) compared to the classification of MCInc vs. MCIc_m6.

**Table 3:** **The LOOCV results using the top 10 combined features**.

**Diagnostic pair**	**ACC (%)**	**SEN (%)**	**SPE (%)**	**AUC**
MCInc vs. MCIc_mixed	66.04	55.26	75.90	0.7346
MCInc vs. MCIc_m6	76.39	65.57	84.34	0.8130
MCInc vs. MCIc_m12	74.66	65.08	81.93	0.7850
MCInc vs. MCIc_m18	73.91	70.51	77.11	0.7729

*ACC, accuracy; SEN, sensitivity; SPE, specificity; AUC, area under the curve*.

By using the top 10 combined features, the features most often selected by the sparse linear regression with the stability selection, we achieved AUC scores in a range between 0.7346 and 0.8130. The features selected (listed in Table 4) show roughly similar features among four diagnostic pairs and include the left inferior parietal cortex (IPC), left frontal pole, left precuneus cortex, left postcentral gyrus, left entorhinal cortex, left MTG, left banks superior temporal sulcus, right caudal middle frontal gyrus, right supramarginal gyrus, right posterior cingulate cortex, right isthmus of the cingulate cortex, and right lingual gyrus. These selected regions have been shown to be related with MCI conversion (Chételat et al., 2005; Fan et al., 2008; Misra et al., 2009; Risacher et al., 2009; Yao et al., 2010; Cai et al., 2015; Kandiah et al., 2015). Moreover, note that nearly all involved network features included the nodal degree (ND).

**Table 4:** **Top 10 combined features selected by the sparse linear regression with stability selection in the LOOCV experiments**.

**Feature**	**Frequency (%)**	**Feature**	**Frequency (%)**
MCInc vs. MCIc_mixed		MCInc vs. MCIc_m12	
CT: IPC_L	100	CT: IPC_L	100
CV: IPC_L	100	CT: cMFG_R	100
ND: MTG_L	100	CV: IPC_L	100
ND: PoCG_L	100	CV: MTG_L	100
ND: LING_R	100	ND: FP_L	100
NL: IPC_L	100	ND: MTG_L	100
CV: SMG_R	99	ND: PoCG_L	100
ND: PCC_R	97	ND: LING_R	100
CV: MTG_L	96	CV: SMG_R	87
ND: IPC_L	47	CT: BSTS_L	63
MCInc vs. MCIc_m6		MCInc vs. MCIc_m18	
CT: IPC_L	100	CT: IPC_L	100
CT: MTG_L	100	CT: MTG_L	100
CV: IPC_L	100	CT: cMFG_R	100
ND: MTG_L	100	CT: PCUN_L	99
ND: LING_R	100	ND: PCC_R	99
CV: MTG_L	99	CT: IstCC_R	98
ND: PoCG_L	99	CT: BSTS_L	90
NL: ENT_L	88	CV: IPC_L	90
CV: SMG_R	85	ND: MTG_L	70
NL: IPC_L	74	CV: MTG_L	53

*CT, cortical thickness; CV, cortical volume; CS, cortical surface area; NL, nodal length path; ND, nodal degree, L, left hemisphere; R, right hemisphere*.

**Figure 3:**
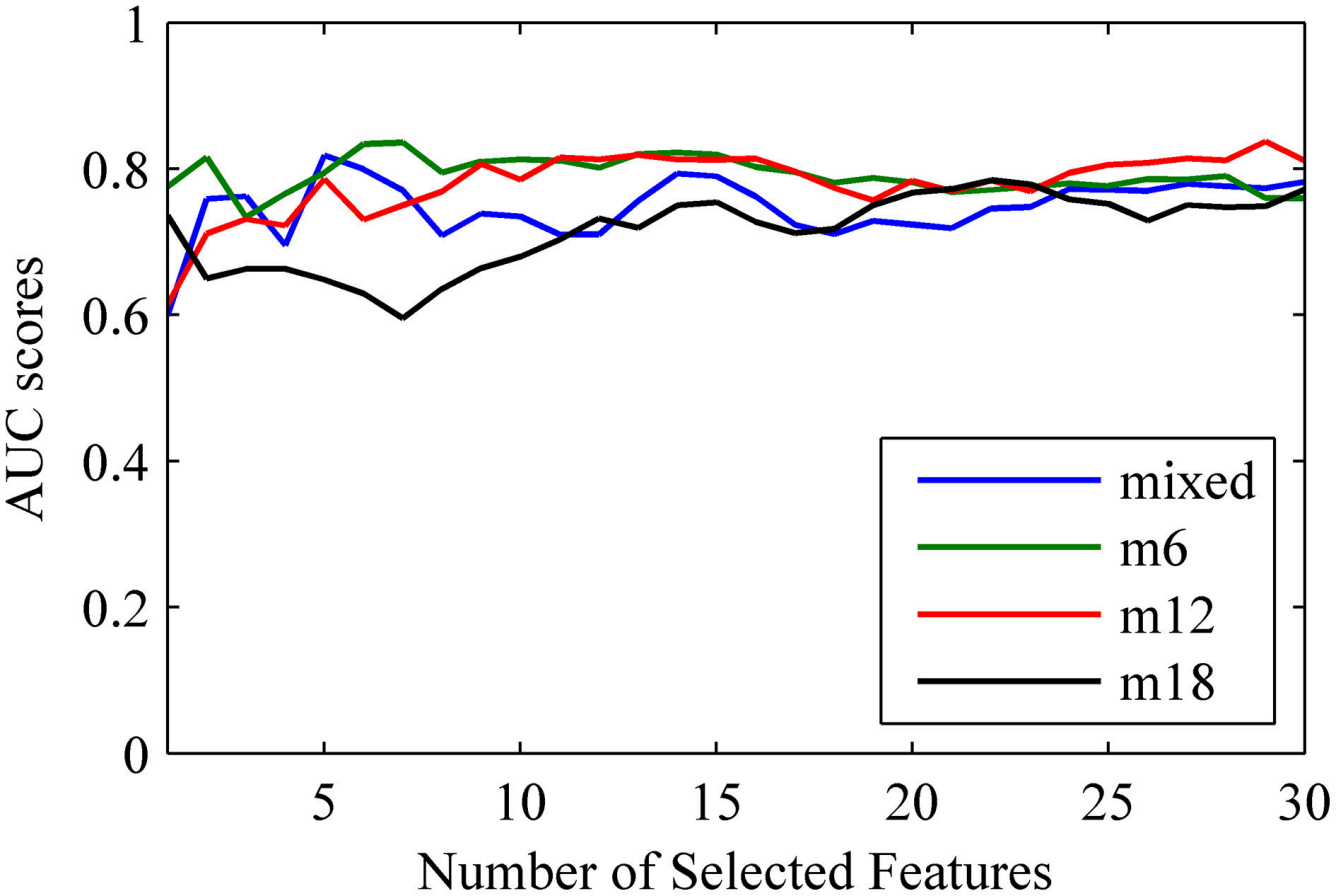
**The change of AUC scores as a function of the number of combined features**.

To demonstrate the impact of the number of selected features, we conducted the classification using the top *K* combined features for *K* = 1, 2, …, 30. The classification performances and AUC scores are depicted in Supplementary Table 1 and Figure 3, respectively. As shown in Figure 3, the AUC stabilizes after the top 12–15 features are included and the best classification results are observed in the classification of MCInc vs. MCIc_m6 and MCInc vs. MCIc_m12.

To examine the added benefit of the network measures, we applied the sparse linear regression with the stability selection to either the MRI or the network measures. The classifier model performances and ROCs are depicted in Table 5 and Figure 2. As shown in Table 5, MRI achieved the best AUC scores (0.8002 for MCInc vs. MCIc_m6), while the network biomarkers performed slightly worse (AUC = 0.6974, 0.6006, 0.7481, 0.6140, for mixed, 6, 12, and 18 months before diagnosis of probable AD, respectively). The top 10 MRI and network features are listed in Supplementary Tables 2, 3. Note that most items in Table 4 and Supplementary Tables 2, 3 match, and that several cortical surface area (CS) features were included in the classifier, only when the signal MRI was used for prediction.

**Table 5:** **The LOOCV results using top 10 MRI features and top 10 network features**.

**Diagnostic pair**	**Top 10 MRI features**				**Top 10 network features**			
	**ACC (%)**	**SEN (%)**	**SPE (%)**	**AUC**	**ACC (%)**	**SEN (%)**	**SPE (%)**	**AUC**
MCInc vs. MCIc_mixed	72.33	68.42	75.90	0.7865	64.78	61.84	67.47	0.6974
MCInc vs. MCIc_m6	75.00	63.93	83.13	0.8002	61.81	49.18	71.08	0.6006
MCInc vs. MCIc_m12	73.29	63.49	80.72	0.7885	70.55	61.90	77.11	0.7481
MCInc vs. MCIc_m18	78.40	45.24	95.18	0.7321	66.40	33.33	83.13	0.6410

*ACC, accuracy; SEN, sensitivity; SPE, specificity; AUC, area under the curve*.

## Discussion

In this study, we established an efficient MCI conversion classification framework using a combination of MRI and network measures. The increased prediction accuracies that we observed suggest that it may be possible to identify conversion from MCI to AD using the combination of MRI and network measures. Moreover, the homogenization of the MCIc sub-groups showed improved classification of the short-term prediction, yielding a more consistent pattern of cortical neurodegeneration.

Our findings show (Tables 3, 5) that the combination of MRI and thickness network measures outperforms either MRI or network measures alone, in the prediction of conversion from MCI to AD. In addition, the results showed that brain morphometric was a better predictor compared with thickness network measures, suggesting abnormalities may exist across different ROIs during the conversion period to AD. Moreover, the increased predictive power of the combined classification methodology suggests that a co-variation of the abnormalities across different regions is necessary for the detection of the early transition from MCI to AD. Without requiring new sources of information, our prediction AUCs are in line with previous studies (Cui et al., 2011; Ye et al., 2012; Eskildsen et al., 2013; Raamana et al., 2015), which used multivariate biomarkers including thickness, thickness network, CSF, and cognitive measures. Cui et al. (2011) showed that with a combination of MRI, CSF, neuropsychological and functional measures (NMs), MCInc vs. MCIc were classified with an AUC of 0.796 at baseline. However, the specificity that was achieved was under 50% (48.28%), despite adding CSF and five NMs measures that have been thought to be useful in conversion prediction. On the other hand, Ye et al. (2012) who used a spare logistic regression with stability selection and a combination of 15 features including MRI, APOE gene, and cognitive measures, achieved the best reported classification results to date with an AUC of 0.8587 (Ye et al., 2012). Our results demonstrate slightly lower accuracy levels, but we only used one source of information and a smaller number of selected features. In addition, obtaining CSF and APOE gene measures may not be applicable for some subjects, and thus make be difficult to obtain during data integration. Eskildsen et al. (2013) have also distinguished MCIc from MCInc at various intervals prior to diagnosis, with AUC scores of 0.809 and 0.762 for MCIc_m6 and MCIc_m12, respectively. Raamana et al. (2015) achieved an AUC of 0.680 using a novel approach that utilizes thickness network fusion measures for the prediction of MCI conversion. Classification results are summarized in Table 6.

**Table 6:** **Comparison of classification performance of different methods**.

**Article**	**Method**	**MCInc/MCIc**	**Scans**	**ACC (%)**	**SEN (%)**	**SPE (%)**	**AUC**
Cui et al., 2011	Multivariate predictors (MRI, CSF, and NM scores)	87/56	baseline	67.1	96.4	48.3	0.796
Ye et al., 2012	SLR+SS (MRI, genetic, and cognitive measures)	177/142	baseline	–	–	–	0.859
Eskildsen et al., 2013	Patterns of cortical thinning	134/122	6 months	75.8	75.4	76.1	0.809
		134/123	12 months	72.9	75.8	70.2	0.762
Raamana et al., 2015	Thickness network fusion	130/56	baseline	64.0	65.0	64.0	0.680
Proposed	Combination of MRI and thickness network	83/76	baseline	66.0	55.3	75.9	0.735
		83/61	6 months	76.4	65.6	84.3	0.813
		83/63	12 months	74.7	65.1	81.9	0.785
		83/42	18 months	73.9	70.5	77.1	0.773

*The best multivariate predictors of MCI conversion are shown for each study*.

*ACC, accuracy; SEN, sensitivity; SPE, specificity; AUC, area under the curve; CSF, Cerebrospinal Fluid; NMs, neuropsychological and functional measures; SLR+SS, sparse logistic regression with stability selection*.

Importantly, the stability selection provides a small subset of discriminative patterns (see Table 4 and Supplementary Tables 2, 3) for effective and efficient screens. Our findings showed that most of the MRI features in the top 10 combined features were cortical thickness and volume. The consistent features that were included in most pairs with a high frequency were the cortical thickness and volume of the left IPC; and the cortical volume of the left MTG and of the right supramarginal gyrus (SMG), suggesting that abnormities in these regions may be important predictors of conversion (Chételat et al., 2005; Pennanen et al., 2005; Fan et al., 2008; Karas et al., 2008; Whitwell et al., 2008; Desikan et al., 2009; Schroeter et al., 2009; Li et al., 2011; Wang et al., 2016). Additionally, we found that the features selected were predominately in the left hemisphere (Table 4). The potential asymmetry is possible related to the disease progression, since the pattern of atrophy in AD was fairly symmetric (Fan et al., 2008). Besides, the selected ROIs were functionally associated with episodic memory (MTG, IPC) and attention (posterior cingulate cortex). Other features that were included were the nodal degree of the left MTG, the right lingual gyrus (LING) and the left postcentral gyrus (PoCG). Previous studies have found that subjects with MCI have abnormal network patterns in the LING and MTG (Yao et al., 2010). In addition, He and colleagues demonstrated an abnormal correlation between bilateral PoCG in AD (He et al., 2008). Moreover, the ROIs selected showed a small overlap between MRI and thickness network, suggesting that informative co-variation of the abnormalities may provide complementary information for classification. Together, our results suggest that changes in the cortical regions may be associated with mechanisms underlying the conversion of MCI to AD, and structural network architecture can be a potential predictor for the classification of imminent conversion.

The classification performances obtained for the MCIc sub-groups showed an improvement when time-homogenization was utilized, which was in line with a previous study (Eskildsen et al., 2013). We found that short-term prediction (6 and 12 months follow up) showed slightly better performances compared with long-term prediction of 18 months (Figure 3). The likelihood for MCIc subjects to be accurately predicted increased with the reduction of conversion prior diagnosis. The small overlap in brain atrophy and network topology, we believe, is the primary reason for improving short-term predictions. Additionally, the relatively low sensitivity for MCInc vs. MCInc_m18 possibly due to the small sample size available to construct the long-term (18 months) classifier model.

On the other hand, we investigates whether the number of features selected influences the classification results. Overall, we found that the AUC scores stabilized after the top nine features were added to the classifier model for the 6 and 12 months follow up. In contrast, for the 18 months follow up, the AUC values increased when the number of selected features was increased, and a strong relationship was observed in the classification of MCInc vs. MCIc_mixed. The stable performances that were observed for the short-term predictions may be attributed to mechanisms associated with the conversion to AD, suggesting more consistent patterns of abnormalities in brain atrophy and network features. The effect of the homogenization of the MCIc patients reveals that predictions are superior when subjects display variable time periods to conversion. Specifically, compared to combined MRI and network features, the top 10 MRI features showed similar performances for short-term predictions, suggesting that the abnormal brain atrophy patterns are strong predictors for short-term prediction. For MCIC_m18 prediction, the sensitivity increased by 25% and the AUC increased by 4%, when we used combined feature sets compared with MRI measures alone, which may indicate that these classes of measures provide complementary information for diagnostic classification. Therefore, informative structural network measures could be potentially useful for classification, especially at the early stage of impairment.

This study has several limitations. One limitation is that there is no consensus regarding the time boundary for MCI converters and MCI non-converters. Another limitation related to network features, is whether the extracted network features reflect characteristics related to AD in an integral and accurate manner. Although several studies (Stam et al., 2007, 2009; He et al., 2008; Yao et al., 2010; Shu et al., 2012; Zhao et al., 2012; Tijms et al., 2014) show that AD and MCI are associated with changes in network properties, there is little agreement about the nature of these changes. Another drawback is that the accuracy of some discriminant classifiers should be interpreted with caution. Future studies are warranted where larger samples and more advanced fusion methods, using more than just node quantitative measurements, may limit overestimation and may overcome direct comparison. Moreover, further studies are needed in order to examine the diagnostic power of the relationship between structural and functional connectivity abnormalities in MCI sub-groups.

## Conclusion

This study investigated the diagnostic power of the combination of MRI and thickness network measures derived from structural MRI to distinguish individuals with MCIc from MCInc. Without requiring new sources of information, our approach shows that the effective combination of MRI and thickness network measures improves the discrimination between MCIc and MCInc, compared with the use of either MRI or network measures separately. Moreover, the selected features are interpretable and are in line with previous findings, and the similar spatial patterns of brain morphometric and structural network alterations are shared among the four groups that we examined. By using longitudinal measures, we also found that short-term prediction shows more stable and better performances compared with long-term prediction. Together, our study provides a new insight into the prediction of MCI to AD conversion, and revealed that structural connectivity is a potential predictor for classification of imminent conversion.

## Author contributions

RW was in charge of the data analysis and manuscript writing. CL helped in speeding up the data analysis. LL helped in calculation and manuscript writing. NF helped was in charge of manuscript verifying. All authors reviewed the manuscript.

## Funding

This research was supported by grants from NSFC (Nos. 61203363 and 61473062), Spanish Ministry of Science and Innovation (PSI2012-34212), the Ramón y Cajal national fellowship program, the 111 project (B12027), and the Fundamental Research Funds for the Central Universities.

### Conflict of interest statement

The authors declare that the research was conducted in the absence of any commercial or financial relationships that could be construed as a potential conflict of interest.
